# Oral Myiasis Caused by Musca domestica Larvae

**DOI:** 10.7759/cureus.80816

**Published:** 2025-03-19

**Authors:** Yassine Akrim, Awatif El Hakkouni

**Affiliations:** 1 Biology Department, Mohammed VI University Hospital, Faculty of Medicine and Pharmacy, Cadi Ayyad University, Marrakech, MAR

**Keywords:** larvae, musca domestica, myiasis, oral cavity, oral hygiene

## Abstract

Myiasis refers to the infestation of human and animal tissues by fly larvae. Oral myiasis is a relatively rare condition typically affecting individuals with low socio-economic status and poor hygiene. This case report describes a 21-year-old male patient with a history of chronic smoking and ischemic stroke, who developed oral myiasis following intubation for glioblastoma surgery. After 10 days post-operation, multiple larvae were identified in the oral cavity. Manual extraction and treatment with metronidazole were performed, leading to initial healing. However, the patient subsequently developed respiratory infection and septic shock, ultimately resulting in death. The report highlights the importance of prevention over treatment, emphasizing individual hygiene and collective measures to control house fly populations and improve living conditions. Effective management of myiasis requires awareness of predisposing factors and timely intervention to avoid serious complications.

## Introduction

Myiasis refers to the infestation of human and animal tissues and organs by fly larvae. While commonly observed in various body parts, including the ears, nose, eyes, paranasal sinuses, lymph nodes, anus, vagina, and oral cavity [1,2]. Oral myiasis is relatively rare. This infrequency is due to the fact that oral cavity tissues are not often damaged and have less contact with the external environment [3]. Oral myiasis primarily affects the periodontium, buccal and palatal mucosa, lips, and tongue [4]. It is more commonly found in humid and warm climates, particularly in developing regions. Several predisposing factors contribute to the risk of oral myiasis, including low socio-economic status, malnutrition, substance abuse, poor oral hygiene, facial injuries, open wounds, habitual mouth-breathing, mental disabilities, aging, and prolonged exposure to external environments [1]. Thirteen different species of flies have been implicated in causing oral myiasis, with *Musca domestica* being one of the most frequently encountered species [5]. There are no established standard procedures for treating oral myiasis, but common approaches include the removal of larvae, sometimes with topical applications of substances like ether, chloroform, olive oil, or iodoform to expel the larvae [6]. Additionally, ivermectin has proven effective in managing oral myiasis [7]. We present the case of an oral gingival myiasis in a young man who was intubated for glioblastoma surgery.

## Case presentation

**Figure 1:**
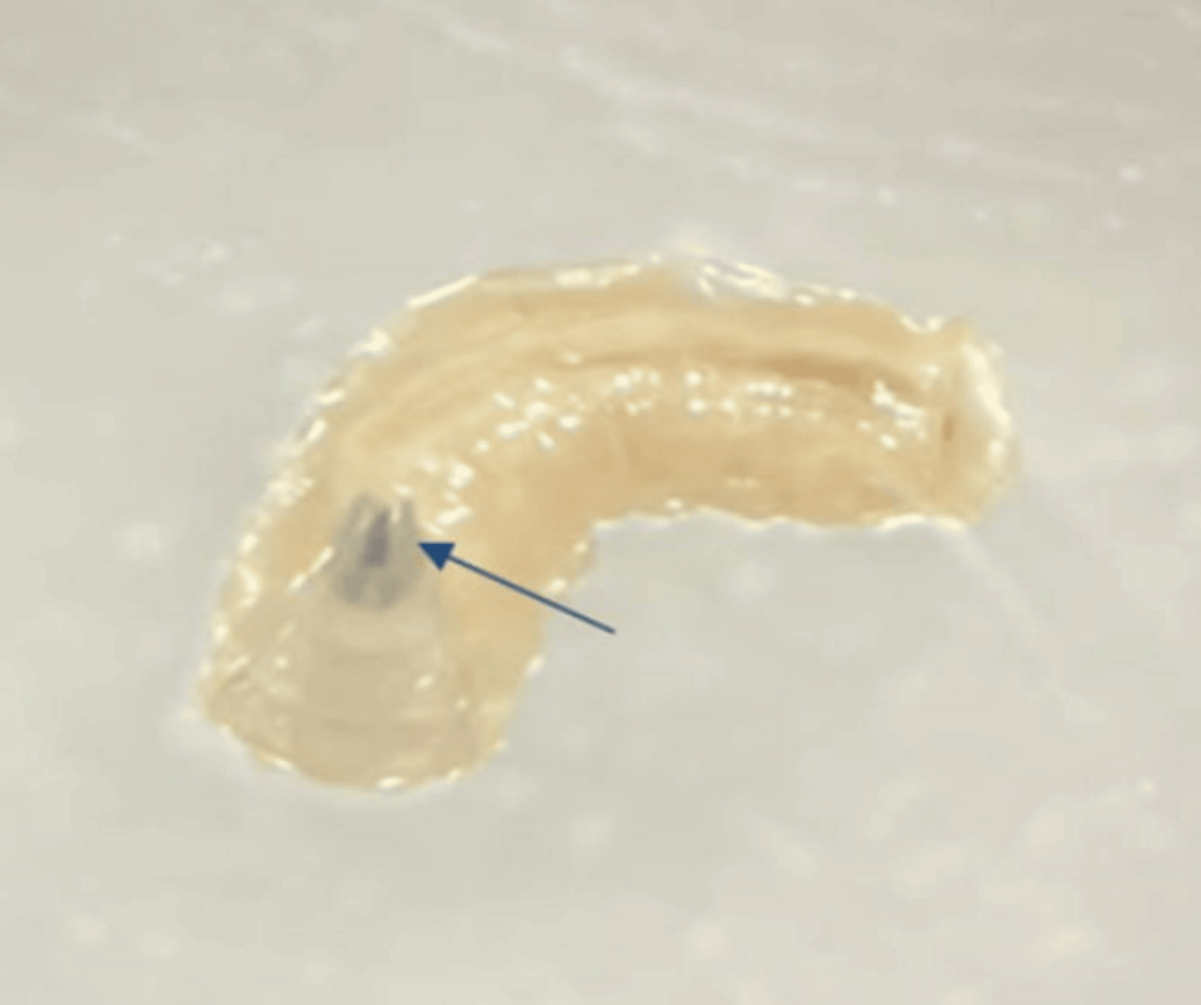
Tapered anterior part of the maggot containing a pair of hooks (arrow).

**Figure 2:**
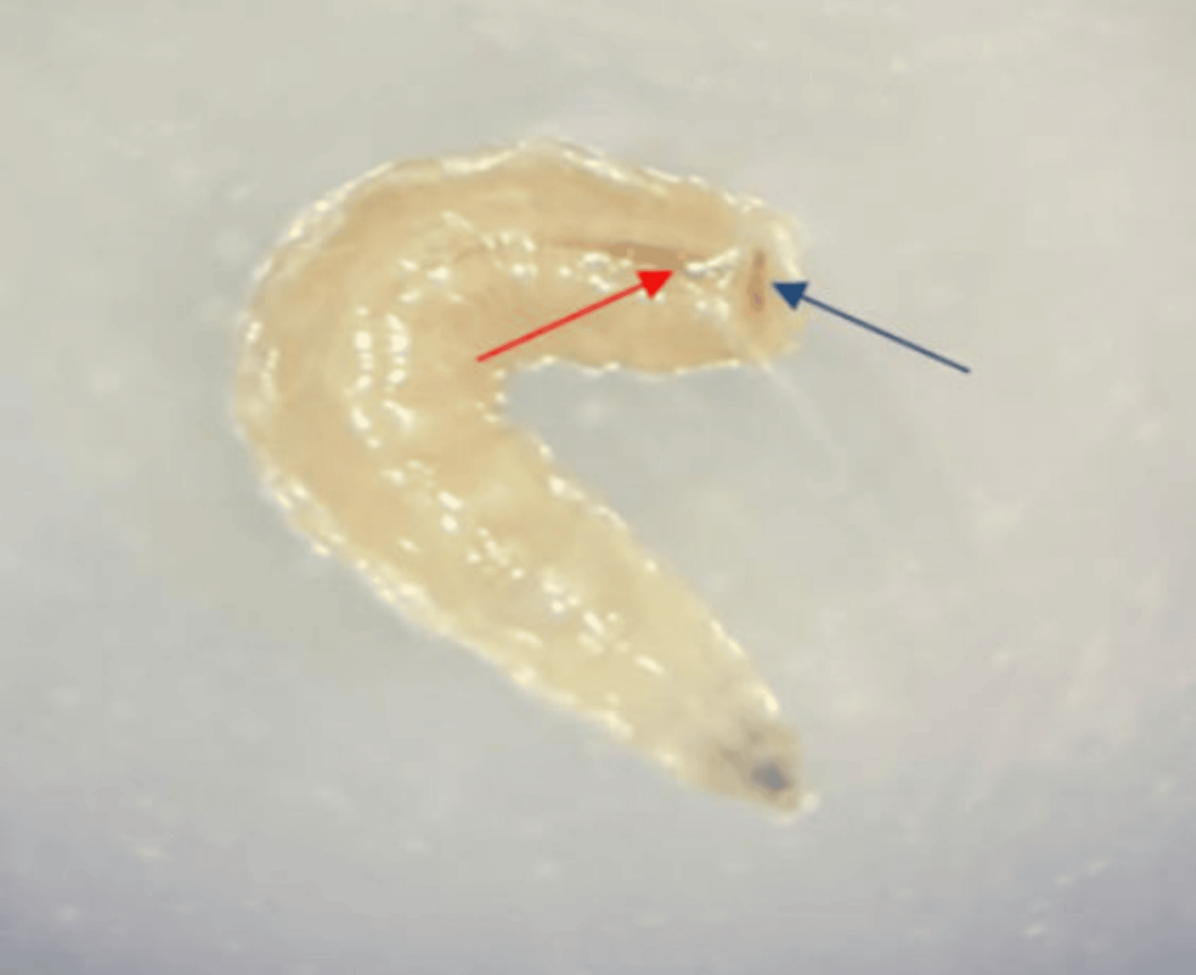
Wide posterior part of the maggot with spiracles (blue arrow) leading to the respiratory system (red arrow).

A 21-year-old male patient with a history of chronic smoking for three years and an ischemic stroke eight months ago was admitted to the hospital for surgical management of glioblastoma. The patient underwent a surgical procedure involving tumor resection and was transferred to the postoperative intensive care unit. In the absence of signs of awakening, the patient remained intubated and artificially ventilated. Intubation was initially performed via the orotracheal route and then via the nasotracheal route. After 10 days post-operation, the healthcare team in the intensive care unit noted the presence of multiple small, sticky white worms in the oral cavity of the intubated patient. The team of the parasitology-mycology department was called upon to identify these specimens. During the intraoral examination, the patient's mouth was open due to intubation, and the oral hygiene was poor. The presence of fetid odor, dental calculus, caries, and periodontal pockets was noted in both the maxillary and mandibular regions. Three maggots were detected in the gingival sulcus around the upper right premolars and molars. The surrounding area was inflamed. The infected area was thoroughly examined. Initially, the larvae were removed by applying pressure around the lesion and using forceps. The site was cleaned and disinfected by irrigating it with a saline solution and antiseptic solution to remove any remaining debris. The examination of the removed specimens under a binocular magnifier revealed cylindrical larvae that were creamy white and approximately 10 mm long, featuring a tapered anterior part with a pair of dark mouth hooks (Figure 1) and a wide flattened posterior part with spiracles leading to the respiratory system (Figure 2). These larvae exhibited a wriggling motion.

Based on these clinical findings, the case was diagnosed as oral myiasis caused by *M. domestica*. The radiological examination did not show any lesions in the bones or in the sinuses. The patient's laboratory tests showed no abnormalities except for a C-reactive protein (CRP) level of 54 mg/L and an elevated white blood cell count of 12,800 cells/mm³, predominantly neutrophils (Table 1).

**Table 1:** Laboratory results: complete blood count (CBC) and C-reactive protein (CRP).

Test	Results	Reference range
Hemoglobin	13.2	12-14 g/dL
White blood cell count	12.8	4-10.5/μL
Neutrophil count	9.7	2-7/μL
Lymphocyte count	1.72	1.5-4/μL
Platelets	225	150-400/μL
C-reactive protein	54	<6 mg/L

After the mechanical extraction of the larvae, the patient received intravenous metronidazole at 1.5 g per day, along with a chlorhexidine mouthwash. A follow-up after 10 days revealed complete healing of the lesion. However, the patient's neurological and respiratory condition deteriorated. He developed a respiratory infection complicated by septic shock, leading to his death.

## Discussion

Myiasis is an infestation of humans and other vertebrates by fly larvae from the Diptera order, which feed on the host's dead or living tissues, bodily fluids, or consumed food. It can manifest in various ways, including infections of the skin, intestines, nasal passages, eyes, and occasionally the oral cavity [8].

Most cases reported in developing countries are attributed to inadequate personal hygiene, poor living conditions, and a warm climate that promotes fly breeding. In this case, the patient had a low socio-economic status and lived in poor conditions. The patient's neurological condition caused his mouth to remain open and exposed to the external environment, which facilitated the laying of eggs inside the mouth, where the infestation developed. Additionally, poor oral hygiene and periodontal diseases created a favorable environment for the larvae to thrive.

*M. domestica*, commonly known as the housefly, lives in close proximity to humans, contaminating food and breeding in garbage and animal waste. The virulence of this insect is associated with the irritation it causes, as well as the indirect harm from its ability to transmit pathogens such as *Salmonella, Shigella, Campylobacter, Escherichia, Enterococcus, Chlamydia*, and various other species that can result in illness. There are two primary routes for maggot infestation in humans. The most common is the direct deposition of eggs on living tissues or mucous membranes of natural body orifices. The second route involves the consumption of contaminated food, particularly among individuals with poor food hygiene practices [9]. The lifecycle of these flies lasts approximately 7 to 10 days, progressing from the egg stage to the larval, pupal, and then adult fly stages. However, under less-than-ideal conditions, the lifecycle can extend to as long as two months. A female fly can lay up to 500 eggs in several batches. In warm weather, the maggot hatches from the egg within 8 to 20 hours. The larva goes through three instars and becomes a fully grown maggot [10]. At appropriate temperatures, the maggots develop by feeding on decomposing tissues, which is why an intermediate host is always required. The larval stage lasts six to eight days, during which they parasitize humans. The larvae have backward-facing hooks that anchor them to the surrounding tissues. They are photophobic and tend to hide deep within the tissues to create a suitable niche for pupation. The presence of these hooks makes manual removal of the larvae from the host difficult. Pupae, which differ in shape from the larvae, complete their development in two to six days at temperatures between 32°C and 37°C [11].

Early management of myiasis is crucial to prevent serious deep tissue damage. Therapeutic principles include manual removal of larvae with or without topical asphyxiating agents (such as ether, calomel, chloroform, olive oil, turpentine, phenol mixture, and iodoform) to encourage larvae expulsion, administration of antibiotics in case of secondary infections, maintaining good local hygiene, and a proper diet. Systemic ivermectin can yield positive outcomes in severe cases of myiasis. In our case, metronidazole was prescribed to prevent secondary infection. The fatal outcome was not attributed to the parasitic infection but rather to septic shock originating from the lungs and neurological failure [8].

On a collective level, preventing human myiasis involves implementing programs to control adult house flies, ensuring cleanliness in living areas, maintaining effective sanitation systems, and promoting health education. On an individual level, it is crucial to emphasize personal hygiene, especially for vulnerable individuals, use mosquito nets to block flies from reaching the body, and apply insecticide sprays in areas where house flies tend to congregate [12].

## Conclusions

*M. domestica, *known for transmitting pathogenic organisms, is also a potential cause of myiasis. Oral myiasis caused by *M. domestica* is a rare condition. It primarily affects individuals with low socio-economic status, poor hygiene practices, and underlying health issues. This case emphasizes the importance of maintaining a high index of suspicion for unusual oral presentations in patients with risk factors for developing oral myiasis. Prevention should be prioritized in such cases, requiring both individual and collective measures rather than focusing solely on treatment.
